# Supplementary material for: Hybrid Models and Biological Model Reduction with PyDSTool
Source: PLoS Comput Biol. 2012 Aug 9;8(8):e1002628. doi: 10.1371/journal.pcbi.1002628 (PMC3415397; doi:10.1371/journal.pcbi.1002628)
Supplement: Text S4 — Complete source code for the PyDSTool package (version 0.88.120504). Includes API documentation and help files linking to web pages. This file is identical to the current public release on Sourceforge.net. (ZIP) [file pcbi.1002628.s004.zip › PyDSTool/html/PyDSTool.Toolbox.optimizers.line_search.fixed_last_step_modifier-pysrc.html]

xml version="1.0" encoding="ascii"?


PyDSTool.Toolbox.optimizers.line\_search.fixed\_last\_step\_modifier


| Home | Trees | Indices | Help | PyDSTool |
| --- | --- | --- | --- | --- |

Package PyDSTool ::
Package Toolbox ::
Package optimizers ::
Package line\_search ::
Module fixed\_last\_step\_modifier

[hide private]

[frames] | no frames]

# Source Code for Module PyDSTool.Toolbox.optimizers.line\_search.fixed\_last\_step\_modifier

```
 1   
 2  # Matthieu Brucher 
 3  # Last Change : 2007-08-28 00:34 
 4   
 5  """ 
 6  Line search decorator that overrides the default alpha_step value with a factor times the last alpha_step 
 7  """ 
 8   


9 -class FixedLastStepModifier(object):


10    """ 
11    Overrides the default step size and replaces it with a factor times the last one 
12    """ 


13 -  def __init__(self, line_search, alpha_factor = 2., **kwargs):


14      """ 
15      Needs to have : 
16        - the decorated line search (line_search) 
17      Can have : 
18        - an alpha modifier, a factor to modulate the last step length (alpha_factor = 2.) 
19      """ 
20      self.line_search = line_search 
21      self.alpha_factor = alpha_factor

22   


23 -  def __call__(self, origin, function, state, **kwargs):


24      """ 
25      Returns a good candidate 
26      Parameters : 
27        - origin is the origin of the search 
28        - function is the function to minimize 
29        - state is the state of the optimizer 
30      """ 
31      if 'alpha_step' in state: 
32        state['initial_alpha_step'] = self.alpha_factor * state['alpha_step'] 
33      return self.line_search(origin = origin, function = function, state = state, **kwargs)

34
```

  


| Home | Trees | Indices | Help | PyDSTool |
| --- | --- | --- | --- | --- |

|  |  |
| --- | --- |
| Generated by Epydoc 3.0.1 on Fri May 4 15:24:15 2012 | http://epydoc.sourceforge.net |
